# The Shared Document as a Tool for Shared Care Planning: A Retrospective Analysis of 160 Cases

**DOI:** 10.3390/healthcare14142211

**Published:** 2026-07-21

**Authors:** Salvatore Simone Masilla, Clara Todini, Barbara Corsano, Andrea Ponzio, Dario Sacchini, Pietro Refolo, Antonio Gioacchino Spagnolo

**Affiliations:** 1 Research Centre for Clinical Bioethics & Medical Humanities, Università Cattolica del Sacro Cuore, Largo F. Vito 1, 00168 Rome, Italy; salvatoresimone.masilla@unicatt.it (S.S.M.); clara.todini@unicatt.it (C.T.); andrea.ponzio02@icatt.it (A.P.); dario.sacchini@unicatt.it (D.S.); pietro.refolo@unicatt.it (P.R.); antoniogioacchino.spagnolo@unicatt.it (A.G.S.); 2 Section of Bioethics and Medical Humanities, Department of Health Care Surveillance and Bioethics, Università Cattolica del Sacro Cuore, Largo F. Vito 1, 00168 Rome, Italy; 3 Fondazione Policlinico Universitario A. Gemelli IRCCS, Largo F. Vito 1, 00168 Rome, Italy

**Keywords:** shared care planning, clinical ethics consultation, advance care planning, shared decision-making, bioethics, multidisciplinary approach, palliative care, informed consent, maternal–fetal medicine, perinatal ethics

## Abstract

Background/Objectives: Shared Care Planning (SCP) represents a collaborative decision-making process integrating advance care planning and shared decision-making to support ethically complex clinical pathways. Since 2016, the Clinical Ethics Consultation (CEC) service at the Fondazione Policlinico Universitario “Agostino Gemelli” IRCCS (FPUG) in Rome has implemented SCP through the use of the Shared Document (SD) for healthcare ethics planning. This study aimed to describe the SD as an operational tool supporting SCP, focusing on its procedural characteristics, multidisciplinary dimension, and the ethical and contextual issues emerging during its implementation. Methods: This single-center, retrospective observational study analyzed digitized medical records of patients who underwent SCP through the drafting of one or more SDs between 2016 and 2024 at FPUG. Data were extracted from SDs and clinical records in accordance with the RECORD guidelines. Variables included processing time, number of meetings with the clinical ethics consultant (CEc), number of healthcare professionals and family members involved, ethical–clinical issues, contextual challenges, and care orientations proposed within the SDs. Descriptive statistics were used to characterize the cohort and operational aspects of the service. Results: Among 454 patients referred to the CEC service, 154 patients underwent SCP, resulting in 160 SDs. The most frequent ethical issues concerned the proportionality of treatments to initiate (70%) and ongoing treatments (31%). Contextual issues emerged in 74% of cases, particularly pregnancy-related situations (44%) and absence of a legal guardian (19%). Drafting of SD required multiple interdisciplinary meetings (mean: 2.4), with an average processing time of 7 days. The number of healthcare professionals involved increased over time, reflecting growing multidisciplinary participation. The most frequent care orientations included palliative care (58%), withholding invasive/intensive maneuvers (34%), and indications for surgical or diagnostic treatments. Conclusions: The SD emerged as a structured clinical–ethical tool supporting complex shared care planning processes beyond issues of informed consent alone. Its use facilitated multidisciplinary deliberation, the integration of ethical and contextual factors, and continuity in care planning across different clinical trajectories.

## 1. Introduction

### 1.1. Shared Care Planning

Shared Care Planning (SCP) is a collaborative decision-making process where patients, family members, and healthcare providers work together to create and implement a personalized care plan tailored to the needs of both the patient and their family. Unlike advance directives, which primarily focus on the patient’s general preferences in potential disease-related or end-of-life scenarios, SCP encompasses all stages of the care journey—from diagnosis to home care, long-term care settings, or hospice environments, including acute care in hospital settings. This patient-centered process aims not only to address clinical and care needs but also to incorporate the patient’s values and preferences.

SCP integrates two classical approaches of modern medicine: advance care planning (ACP) and shared decision-making (SDM). The former, as defined by Rietjens et al. (2017), offers patients the opportunity to reflect on the meanings and consequences of specific pathological scenarios to define goals and preferences regarding future treatments [1]. In this context, ACP “encourages individuals to identify a personal representative and to record and regularly review any preferences, so that their preferences can be taken into account should they, at some point, be unable to make their own decisions” [1]. On the other hand, SDM was defined by the American Medical Association in 2012 as “a formal process or tool that helps physicians and patients work together to choose the treatment option that best reflects both medical evidence and the individual patient’s priorities and goals for his or her care” [2].

In Italy, SCP is regulated under Article 5 of Law 219/2017, titled “Norme in materia di consenso in-formato e di disposizioni anticipate di trattamento”. This law states, “Within the relationship between the patient and the physician, […] with regard to the evolution of the consequences of a chronic and disabling disease, or of a condition characterized by an unstoppable progression and a poor prognosis, shared care planning may be carried out between the patient and the physician. The physician and the healthcare team are required to adhere to such planning should the patient come to be in a condition in which he or she is unable to express consent or lacks decision-making capacity” (translation by the author) [3]. Specifically, the law promotes this practice as a solution to the issue of patients losing the ability to express consent to treatment when already affected by a progressively worsening condition.

### 1.2. Shared Document for Healthcare Ethics Planning

At the Fondazione Policlinico Universitario “A. Gemelli” (FPUG) in Rome, since 2016, the bioethicists of the Clinical Ethics Consultation (CEC) service have been using the Shared Document for healthcare ethics planning (SD) as an operational tool for implementing SCP. Spagnolo et al. (2019) describe the standard operating procedures of the CEC service at FPUG [4], stating that CEC can take two forms depending on the circumstances: a summary document of the consultation, which is attached to the patient’s medical record, or an SD, particularly when the ethical–clinical decision concerns not a specific treatment but a global care plan to be offered to the patient. Shared decision-making is typically initiated by the treating multidisciplinary team or following input from a clinical ethics consultant when prospective care planning is required. In contrast, a CEC is generally requested to support the resolution of a specific ethical issue, whereas SD is intended to facilitate an ongoing process of shared planning with the patient and/or family in anticipation of possible future clinical scenarios. The latter requires the shared involvement of the patient and/or family members and is signed by all stakeholders involved in the case before being included in the medical record [4,5,6].

Corsano et al. (2021) affirm that the SD represents the outcome of a shared decision-making process with the patient [7]. On one hand, it operationalizes the principle of beneficence/non-maleficence inherent in medical practice. On the other hand, it fully respects the principle of patient autonomy, which is not seen as solitary autonomy but rather as autonomy supported and embedded within the physician–patient relationship. SCP leads to the development of a care plan that provides the most appropriate care, considering the clinical and environmental context [7].

The SD is drafted following a multidisciplinary meeting where the care team presents the clinical case, highlighting challenges related both to treatment (clinical condition, comorbidities, prognosis, etc.) and contextual aspects (family dynamics, patient adherence, disagreements within the team, communication barriers, etc.). During this meeting, the care team also specifies the question for which the CEC has been requested. Concurrently, the team evaluates the need for SCP and the drafting of an SD to accompany the patient’s care path.

Following the multidisciplinary meeting and discussions (often involving multiple meetings) with the patient and their family, a preliminary draft of the SD is prepared. If the patient has lost decision-making capacity and no legal representative has been designated, family members are involved to reconstruct the patient’s wishes, and they are given access to the SD.

The draft, written by the clinical ethics consultant (CEc), is then shared with the participants of the multidisciplinary meeting for potential revisions, as well as with the patient, their legal representative, and, if applicable, family members. The final version of the document is signed by all involved parties.

The SD does not follow a fixed structure but is tailored to the specific circumstances of the case. However, several recurring sections characterize the document, as outlined in the Section 3.2.

### 1.3. The CLEAR Study

The study titled “Clinical Ethics Consultation at the Patient’s Bedside: A Retrospective Observational Study on the Experience of the Clinical Ethics Consultation Service at the Fondazione Policlinico Universitario ‘Agostino Gemelli’ IRCCS—Rome (CLEAR)” was approved by the Territorial Ethics Committee “Lazio Area 3” on 16 October 2025. The study was conducted by the Research Center for Clinical Bioethics and Medical Humanities (CRiBCeMH) at the Università Cattolica del Sacro Cuore in Rome.

This retrospective observational study analyzed the activity of the CEC service at the Policlinico Universitario “Agostino Gemelli” IRCCS in Rome (FPUG) between 2016 and 2024, following the integration of the service into the hospital information system, which enabled improved tracking of CEC requests. The present paper focuses on a specific subgroup of the study population, namely patients for whom SCP was developed through the CEC service using an SD.

## 2. Objectives

The aim of this study was to describe the SD as an operational tool to support SCP, illustrating its procedural characteristics, multidisciplinary implementation, and the clinical, ethical, and contextual issues that emerged during its use in routine practice.

## 3. Materials and Methods

This work is based on data from a single-center, retrospective study (CLEAR) conducted on the medical records of FPUG patients for whom an SD was drafted and therefore underwent SCP between 2016 and 2024. All analyzed cases received support from the Clinical Ethics Consultation service, operating in collaboration with the Bioethics and Medical Humanities Section of the Department of Safety and Bioethics at Università Cattolica del Sacro Cuore in Rome.

This study was reported in accordance with the RECORD guidelines proposed for studies based on routine data collection [8].

### 3.1. Population

Between 2016 and 2024, 454 patients were referred for clinical ethics consultation at FPUG. Among these, 154 patients underwent SCP through one or more SDs, totaling 160 SDs (as five patients had more than one document drafted). This study analyzes data related to this latter group of patients. Descriptive statistics indices were used to describe the cohort in terms of age, gender, clinical conditions, prognosis, presence of life-sustaining treatments, capacity to express informed consent, and the clinical area.

The clinical condition at the time of CEC was classified into five categories based on the overall severity of the patient’s status as documented in the medical record. ‘Good’ indicated a stable clinical condition without evidence of immediate life-threatening impairment; ‘Fair’ indicated the presence of clinically relevant disease requiring medical management but with overall physiological stability; ‘Serious’ referred to patients with significant systemic compromise and a high risk of clinical deterioration; ‘Critical’ identified patients with life-threatening conditions requiring intensive monitoring and/or advanced life-support interventions; and ‘Terminal’ referred to patients with irreversible end-stage disease in whom death was expected despite ongoing treatment.

Prognosis was categorized according to the expected short- to medium-term clinical outcome as assessed by the attending clinician. ‘Good’ indicated a high likelihood of recovery or stabilization; ‘Guarded’ indicated uncertainty regarding the outcome because of disease severity or the presence of significant comorbidities; ‘Severe’ indicated a low probability of recovery and a high risk of persistent major morbidity or death; ‘Poor’ referred to patients with an expected fatal outcome or an extremely limited likelihood of meaningful recovery.

### 3.2. Chart Review: Variables and Data Source

Data from digitized medical records in FPUG’s Information System were consulted following approval by the Territorial Ethics Committee. This allowed the comparison of data from the anamnesis summary in the SD with those recorded in the clinical diary. The SD itself, stored in the medical record, was analyzed to extract information regarding the ethical questions driving multidisciplinary reflection, contextual challenges complicating the decision-making process, and the recommendations shared with patients and, in some cases, family members.

Additional data (processing time, number of CEc meetings, number of signatories, and family involvement) were extracted to describe the intervention methodology adopted by the CEC service during the study period to support SCP processes.

Although SDs lack a fixed structure, they all share common elements, including the following:

(a) Document details: header, date, document type (CEC summary or SD), clinical unit;
(b) Patient demographics: name, surname, age, and nosological code;
(c) Initial access data: date, description of the episode or pathology, clinical conditions;
(d) Anamnesis summary;
(e) Treatments administered during hospitalization (for inpatients): list of diagnostic tests, treatments, and specialist visits;
(f) Description of the patient’s current condition: clinical conditions, ongoing treatments, presence of catheters or life-sustaining treatments, communication capacity, and compliance;
(g) Summary of the multidisciplinary meeting: request and execution dates, list of attendees, emerging themes, challenges (contextual or family-related, communication barriers, etc.), treatment scenarios, prognosis;
(h) Shared orientations;
(i) Signature spaces.

To provide a descriptive overview of the service and its operational characteristics, the raw data contained in the SDs were systematically processed and categorized in order to generate indicators useful for describing the SCP process and the activities of the CEC service. In particular, the following variables were analyzed:

(a) Processing time [9]: number of days between the CEC request and the signing of the SD. This indicator was selected to evaluate the temporal dimension of the SCP process and to assess the responsiveness and operational efficiency of the service in supporting complex care planning.
(b) Number of meetings with the CEc before the SD was finalized [10]. This variable was included to describe the level of interaction required during the decision-making process and to explore the complexity and intensity of ethical support provided before the formalization of the SCP.
(c) Number of HCPs involved in the SCP and signing the SD [11]. This indicator was chosen to assess the degree of multidisciplinary involvement in the SCP process, as shared care planning often requires the contribution of multiple healthcare professionals with different clinical and ethical competencies.
(d) Number of family members involved in supporting the SCP [12]. This variable was analyzed to evaluate the extent of family participation in the SCP pathway, considering the central role that relatives and caregivers may play in supporting patients’ preferences, communication, and shared decision-making processes.

In addition, qualitative items extracted from the SDs were analyzed, including ethical–clinical issues, contextual issues, and the orientations proposed by the team. These variables were selected to explore the ethical dimensions underlying SCP requests, the contextual factors influencing clinical decision-making, and the types of ethical guidance provided during the consultation process.

These variables (including clinical condition and prognosis) reflected the assessment of the treating clinical team at the time of SD preparation. In cases where the SD did not explicitly report the clinical condition or prognosis, researchers reviewed the available medical records to identify the relevant clinical information and complete the data extraction. Ethical and contextual issues were coded based on the information documented in the SD records and associated clinical documentation.

### 3.3. Risk of Bias

A potential bias in evaluating data related to the patients’ clinical conditions is the consistent involvement of the CEc in the Perinatal Hospice team. This team supports mothers and families facing complex pregnancies with pathological conditions. However, under the current operating system, direct patient care is focused on the mother, who is recorded as the patient. Thus, pregnancy is highly prevalent (70 out of 160 cases). In the report, the mothers’ clinical conditions (50% categorized as ‘good’ and the remainder as ‘fair’) may appear inconsistent with the SD orientations, which often address the unborn child. For example, some SDs indicate palliative care or withholding intensive or invasive interventions in case of acute events. These indications pertain to the fetus rather than the mother. Additional sources of bias should also be acknowledged, including potential abstractor bias during the retrospective chart review and temporal changes in documentation practices over the 8-year study period. These potential sources of bias were mitigated by the use of a standardized data extraction form with predefined variables and by independent double data extraction (performed by SSM and CT), which enhanced the consistency and reliability of data collection across the study period.

### 3.4. Statistical Methods

The cohort identified among patients for whom an SD was drafted from 2016 to 2024 was described based on the following data: gender, clinical conditions at the time of CEC, prognosis, capacity to provide informed consent, clinical area, presence of life-sustaining treatments, presence of a legal guardian, and specific contextual or family-related challenges [10,13].

Operational strategy data (processing time, number of signatories, and family involvement) were reported using simple descriptive statistics indices.

Finally, a descriptive analysis of the relative frequencies of ethical questions requiring the support of a clinical ethics consultant was conducted. Orientations reported in the SD were analyzed and related to patients’ clinical conditions (good, fair, serious, critical, and terminal) and their overall prognosis (good, severe, poor, and guarded).

Percentages are consistently calculated using the total number of SDs (*n* = 160) as the denominator. The only exception is the gender distribution reported in Table 1, where percentages are calculated based on the total number of patients (*n* = 154).

## 4. Results

### 4.1. Patient Data

Of the 454 patients referred to the CEC service between 2016 and 2024, 154 were involved in SCP, resulting in a total of 160 SDs. The average age of patients was 38 years (median: 36 years), ranging from 2 days to 90 years. 52% of patients were between 20 and 44 years old. Of the total, 72.6% were female, and 27.4% were male.

Notably, 46% of patients were unable to provide informed consent, and among these, 31 patients did not have a legally appointed guardian at the time of SCP formulation. This issue was addressed within the SD by facilitating the appointment of a guardian via a request to the guardianship judge, often with support from the department team.

At the time of SD drafting, patients’ clinical conditions were distributed as follows (see Table 1): good (23%), fair (23%), serious (23%), critical (3%), and terminal (29%). Regarding prognosis, the following parameters were observed: good (41%), severe (5%), guarded (9%), and poor (45%).

These prognosis data require further clarification because 61 patients at the time of SD drafting were pregnant, with fetuses affected by congenital or life-limiting conditions necessitating a structured therapeutic pathway. For these patients, the prognosis was always ‘good’ (which accounts for the high percentage of favorable prognoses among the total patients analyzed). Regarding the fetuses affected by SD drafting, 25 had a poor prognosis, 34 a guarded prognosis, and 2 a severe prognosis.

The clinical areas involved (see Table 1) were as follows: Intensive Care Unit (3%), Internal Medicine and Infectious Disease (15%), Neurology (18%), Pediatrics and Neonatology (22%), Reproductive and Maternal–Fetal Medicine (43%), and Surgery (1%).

### 4.2. Operational Strategy Data

The number of meetings and interdisciplinary discussions involving the CEc before drafting the SD ranged from 2 to 5. 38% of cases required more than two meetings, including interdisciplinary discussions and consultations with the patient or family (average: 2.4; median: 2).

The time required to draft an SD ranged from 60 days to 1 day after the consultation request by the care team, with an average of 7 days and a median of 2 days. These data can be compared to the processing time of simple CEC during the same study period: the time required to issue a CEC report ranged from 0 days (same day as the CEC’s request) to a maximum of 19 days, with an average of 1.1 days and a median of 0 days (67% of CECs were reported the same day as the request).

The number of HCPs signing an SD, reflecting their participation in the multidisciplinary process, ranged from 2 to 18 signatories, with an average of 7.6 and a median of 7. Table 2 reports the frequency of signatures by each HCP in the analyzed cases. The average number of HCPs involved increased from 4.5 in 2016 to 8.6 in 2024.

In 50% of cases, one family member was involved; in 45%, more than one family member (up to a maximum of 4) was involved, and in only 4% of cases, no family member was involved (average: 1.6; median: 1).

The proportion of CEC resulting in an SD increased from 25% in 2016 to 48% in 2024.

### 4.3. Ethical Questions and Contextual Issues

Table 3 shows the absolute and relative frequency of ethical questions requiring CEC support (37% of cases involved more than one ethical question): “Proportionality of treatments to initiate” (70%), “Proportionality of ongoing treatments” (31%), “Proportionality of invasive or intensive treatments in case of acute events” (24%), “Refusal of treatment” (8%), “Off-label treatment” (1%), and “Assessment of care setting” (16%).

In 74% of cases, ethical questions were associated with critical issues (see Table 4) requiring interdisciplinary team support: “Physician–patient communication” (11%), “Difficulty with treatment adherence” (3%), “Pregnancy” (44%), “Medico-legal” (1%), “Lack of a required guardian” (19%), “Social/family issues” (9%), and “Altered state of consciousness” (18%).

### 4.4. Orientations

18 SDs (11%) acquired the patient’s advance directives expressed before the situation requiring SD drafting: 7 patients (4%) provided written dissent to treatments, 10 patients (6%) orally expressed refusal of treatment, and only one patient (0.6%) submitted an advance directive document. 45 SDs (28%) considered religious requests from patients or involved family members, all relating to the administration of sacraments.

**Figure 1:**
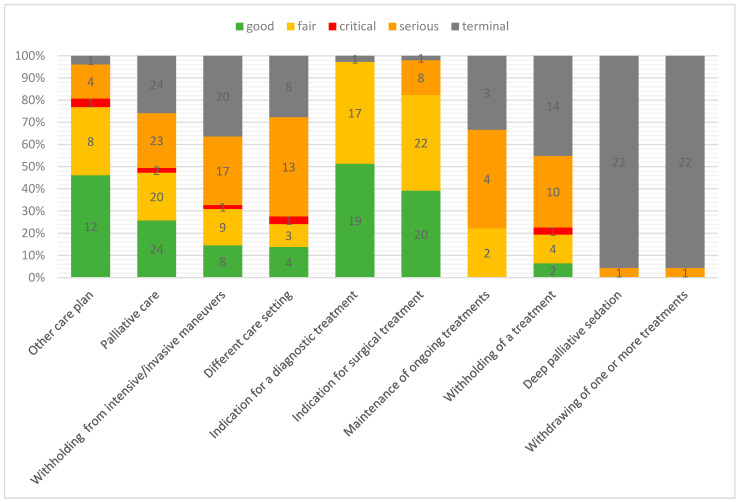
Orientations across different clinical conditions.

**Figure 2:**
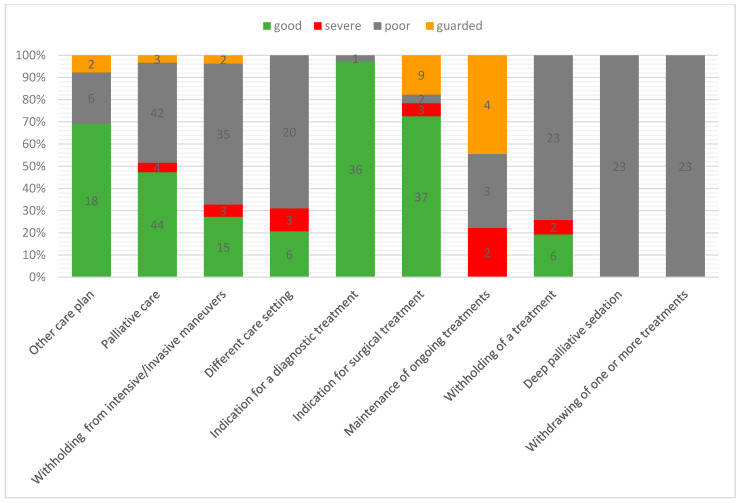
Orientations across different prognoses.

Figure 1 and Figure 2 report the frequency of orientations in SDs based on patients’ clinical conditions and prognoses.

The number of recommendations per SD ranged from 1 to 5 (average: 2.4; median/mode: 2), with 86% of SDs containing more than one recommendation. Below are the identified orientations:

(a) Deep palliative sedation: 23 cases (clinical conditions: 96% terminal, 4% serious; prognosis: 100% poor).
(b) Withdrawing one or more treatments: The distribution of clinical conditions and prognostic categories was the same as observed for deep palliative sedation, although treatment withdrawal was not always associated with palliative sedation.
(c) Withholding treatment: 31 cases (clinical conditions: 45% terminal, 32% serious, 3% critical, 13% fair, 7% good; prognosis: 74% poor, 7% severe, 19% good).
(d) Maintenance of ongoing treatments: 9 cases (clinical conditions: 33% terminal, 44% serious, 22% fair; prognosis: 44% guarded, 33% poor, 22% severe).
(e) Indication for surgical treatment: 51 cases (clinical conditions: 2% terminal, 16% serious, 43% fair, 39% good; prognosis: 18% guarded, 4% poor, 6% severe, 72% good).
(f) Indication for diagnostic treatment: 36 cases (clinical conditions: 3% terminal, 47% fair, 53% good; prognosis: 3% poor, 97% good).
(g) Transfer to a different care setting: 29 cases (clinical conditions: 28% terminal, 45% serious, 3% critical, 10% fair, 14% good; prognosis: 69% poor, 10% severe, 21% good).
(h) Withholding invasive/intensive interventions: 55 cases (clinical conditions: 36% terminal, 31% serious, 2% critical, 16% fair; prognosis: 64% poor, 5% severe, 27% good—in these cases, the good prognosis concerned the pregnant mother, and the indication reported on the SD was related to the fetus in terminal clinical conditions).
(i) Palliative care: 93 cases (clinical conditions: 26% terminal, 25% serious, 22% fair, 26% good; prognosis: 3% guarded, 44% poor, 4% severe, 47%—in these cases, the good prognosis concerned the pregnant mother, and the indication reported on the SD was related to the fetus in terminal clinical conditions).
(j) Other care plans: 26 cases (clinical condition: 4% terminal, 15% serious, 4% critical, 31% fair, 46% good; prognosis: 8% guarded, 23% poor, 69% good).

## 5. Discussion

### 5.1. Operational Strategies

The drafting of an SD involves multiple meetings between the CEc and physicians and/or patients/family members (ranging from a minimum of 2 to a maximum of 5). The time required to draft the document from the initial request by the department averages 7 days. Compared with the mean reporting time for a standard CEC consultation (1.1 days), these data highlight the complexity of SD drafting, which is strongly influenced by the involvement of multiple stakeholders in the decision-making process. This finding is consistent with the international literature on shared decision-making (SDM) and advance care planning (ACP), which identifies iterative and multidisciplinary dialogue as essential components of ethically sound care planning [14].

The SD serves as a synthesis of a therapeutic pathway rather than focusing on a single treatment decision. In fact, the number of orientations included ranges from a minimum of 1 to a maximum of 5, with an average of 2.4 orientations per SD (median 2), and 86% of SDs contain more than one orientation. This multidimensional approach reflects the concept of “relational autonomy,” according to which clinical decisions emerge from ongoing interactions among patients, families, and healthcare professionals [15].

The increase in the number of team members (from 4.5 in 2016 to 8.6 in 2024), the diversity of specializations involved in interdisciplinary meetings (see Table 2), and the consistent presence of family members (only 4% of SDs were written without the involvement of at least one family member) align with previous data regarding the complexity of the service. International studies have demonstrated that interdisciplinary ethics consultations improve communication quality, reduce decisional conflict, and support ethically coherent treatment pathways [16].

### 5.2. What Drives the Request for an SD?

All cases involve at least one ethical/clinical issue, with a high prevalence of discussions regarding the proportionality of treatments to be initiated (70%) or ongoing treatments (31%) (see Table 4). Similar concerns have been widely reported in the literature on end-of-life ethics and shared care planning, where proportionality and appropriateness of treatment represent central ethical criteria in decision-making processes [17,18].

The presence of contextual issues in 74% of cases cannot be considered the sole driver for requesting an SD, given that 26% of cases reported no contextual problems. Nevertheless, contextual complexity remains highly relevant, since these situations often require the contribution of psychologists, social workers, ethicists, or risk managers, increasing the need for multidisciplinary coordination. Studies on clinical ethics consultation have consistently emphasized the importance of contextual and relational dimensions in ethically difficult cases [19].

Regarding issues related to the capacity to provide consent, 74 patients (46%) were already unable to express consent at the time of SCP drafting. Of these, only 31 cases (29% of the total) presented critical issues due to the absence of a legally appointed guardian. Additional consent-related concerns emerged from difficulties in physician–patient communication (11%), compliance issues (3%), and altered states of consciousness (18%). These findings align with the literature on ACP, which highlights the importance of anticipatory planning in situations characterized by cognitive decline, uncertainty, or impaired decisional capacity [1,20].

Thus, current and future consent-related issues represent only one component of the motivations leading healthcare professionals to request an SD. More broadly, the document appears to function as a structured ethical and organizational tool responding to complex clinical, relational, and contextual needs.

### 5.3. The SD as Shared Care Planning

The SD serves not only as a summary of treatments already performed but also as a framework for future care orientations planned by the multidisciplinary team together with the patient and/or family members. The average number of orientations present in each SD (2.4) reflects this prospective and dynamic dimension of care planning. This approach is consistent with international models of ACP and SDM, which conceptualize care planning as a continuous communicative process rather than a single clinical decision [14,21].

Figure 1 and Figure 2 present data on orientations within SDs stratified by prognosis and clinical conditions. Some orientations—such as maintenance of ongoing treatments, indications for surgical interventions, diagnostic procedures, or transfer to alternative care settings—appear relatively independent of prognosis or clinical condition. Others, including deep palliative sedation and withdrawal of treatment, are strongly associated with prognosis and disease severity. International ethical guidelines similarly recognize that decisions regarding treatment limitation and palliative sedation are closely linked to prognosis, symptom burden, and goals of care [22].

Palliative care itself, despite the presence of cases involving patients with favorable prognoses, appears across patients with different prognostic trajectories. This observation supports contemporary literature emphasizing that palliative care should not be restricted to end-of-life situations but integrated early into the management of serious illness alongside curative or life-prolonging treatments [23].

## 6. Limitations

The manuscript’s limitations are the same as those of a single-center, retrospective observational study: the lack of a comparison group, the lack of clinical and qualitative outcome measures, and therefore the inability to evaluate the efficacy of CEC or SD. These outcomes are currently being investigated in other studies, such as the qualitative VALUE study [24] sponsored by the same research center.

The CEC service at FPUG remains an on-demand service, depending on healthcare professionals’ awareness, training, and perceived need for decision-making support. In some areas (e.g., maternal–infant care), the presence of the CEc has grown over time (see Table 1), fostering increasing engagement with the service in which the team has been trained (probably) thanks to the CEc, increasing awareness and leading to a greater number of requests (this remains an unverified hypothesis). In other areas, however, for certain clinical practices (e.g., discontinuing one or more treatments), the team’s decision-making ability has become more autonomous (probably) due to the training provided by the service over the years, leading to a reduced frequency of CEc support requests (although this interpretation remains speculative). Regardless of these hypotheses, it would be incorrect to assume that departments where the CEc is less present have a lower need for decision-making support, as the variables influencing this outcome are too numerous and currently not rigorous enough for objective analysis. Therefore, this study focuses on the quantitative and procedural aspects of what has been done.

Another limitation is obtaining outcome data for a comprehensive evaluation of the service in terms of efficiency, effectiveness, and stakeholder satisfaction [13,24,25].

## 7. Conclusions

A central finding emerging from this study is the ethical, clinical, and contextual complexity underlying the decision to draft an SD. Although Shared Care Planning (SCP) in Italy is formally regulated within the legal framework of informed consent and patient autonomy, the experience described here demonstrates that the SD has progressively evolved into a broader clinical–ethical instrument, capable of addressing not only issues related to consent but also relational, organizational, and contextual dimensions of care. In this sense, the SD appears to function as a structured process of shared deliberation rather than as a merely legal or procedural document.

The analysis of care recommendations in relation to prognosis and clinical condition further supports this interpretation. Our findings suggest that SD is used not only in end-of-life situations or in patients with severe clinical conditions. Rather, the SCP process appears to promote a more comprehensive reflection on the overall goals of care, integrating clinical data with ethical considerations, contextual factors, and the values and preferences expressed by patients and family members. This multidimensional approach is consistent with international literature on shared decision-making and advance care planning, which emphasizes the importance of relational autonomy, interdisciplinary dialogue, and individualized care pathways.

The findings also indicate that the most consistent reason for the use of SDs over time has been the complexity of the therapeutic process itself. Many clinical situations require multiple sequential decisions involving different treatment options, changes in goals of care, and continuous reassessment of proportionality and appropriateness. In this context, the SD provided a framework capable of supporting not only single decisions but also the continuity and coherence of the entire therapeutic pathway.

Beyond its documentary function, the findings of this study suggest that the SD may have the potential to facilitate communication, foster transparency, and support the development of therapeutic alliances among healthcare professionals, patients, and family members. Future studies should further investigate the impact of SDs on clinical outcomes, decisional conflict, patient satisfaction, and the quality of end-of-life care, as well as their potential applicability in other complex care settings.

## Figures and Tables

**Table 1:** Overall demographics and characteristics of the study population.

	#	%
average age (range)	38 years (0–90)	
Male	43	* 28%
Female	111	* 72%
**Clinical conditions**		
Good	36	** 23%
Fair	36	** 23%
Serious	37	** 23%
Critical	5	** 3%
Terminal	46	** 29%
**Prognosis**		
Good	66	** 41%
Severe	8	** 5%
Guarded	14	** 9%
Poor	72	** 45%
**Life-sustaining treatments**		
Yes	79	** 49%
No	81	** 51%
**Capacity**		
Yes	86	** 54%
No	74	** 46%
**Clinical Area**		
Intensive Care Unit	5	** 3%
Internal Medicine and Infectious Disease	22	** 15%
Neurology	29	** 18%
Pediatrics and Neonatology	35	** 22%
Reproductive and Maternal-Fetal Medicine	68	** 43%
Surgery	1	** 1%

* On the total number of patients (154); ** on the total number of SDs (160).

**Table 2:** HCPs involved.

	#	% *
Care providers	160	100%
Clinical ethics consultants	160	100%
Medical consultants	121	76%
Obstetricians	41	26%
Psychologists	76	48%
Nurses	15	9%
Other **	9	2%

* On the total number of SDs (160); ** risk manager, physiotherapist, social worker, spiritual assistant, and continuity of care officer.

**Table 3:** Ethical issues.

	#	% *
Proportionality of treatments to initiate	112	70%
Proportionality of ongoing treatments	49	31%
Proportionality of invasive or intensive treatments in cases of acute events	38	24%
Refusal of treatment	13	8%
Off-label treatment	2	1%
Assessment of care setting	25	16%

* On the total number of SDs (160).

**Table 4:** Critical issues.

	#	% *
No further critical issues	42	26%
Physician–patient communication	17	11%
Difficulty with treatment adherence	4	3%
Pregnancy	70	44%
Medico-legal	2	1%
Lack of a required guardian	31	19%
Social/family issues	14	9%
Altered state of consciousness	29	18%

* On the total number of SDs (160).

## Data Availability

The data presented in this study are not publicly available due to privacy and ethical restrictions involving sensitive clinical information. Aggregated and anonymized data may be available from the corresponding author upon reasonable request and subject to approval by the relevant Ethics Committee.

## Abbreviations

The following abbreviations are used in this manuscript:

CEC	Clinical Ethics Consultation
CEc	Clinical Ethics Consultant
FPUG	Fondazione Policlinico Universitario “A. Gemelli” IRCCS
ACP	Advance Care Planning
SDM	Shared Decision-Making
SCP	Shared Care Planning
SD	Shared Document for Healthcare Ethics Planning
